# Cushioning Performance of the Biomimetic Cobweb Cushioning Silicone Pad

**DOI:** 10.3390/biomimetics8030276

**Published:** 2023-06-28

**Authors:** Changyu Liao, Ye Tian, Wei Xu, Jiahang Zhang, Zhihui Sun, Zhuang Liu

**Affiliations:** School of Light Industry, Harbin University of Commerce, Harbin 150000, China; llz18756980125@163.com (C.L.); hljhrbxw@163.com (W.X.); zjhang15545175157@163.com (J.Z.); sunzhihui62@163.com (Z.S.); hitprint@163.com (Z.L.)

**Keywords:** biomimetic structure, silicone, 3D printing, cushion pad

## Abstract

At present, the packing method of “plastic bag–buffer packing–packing paper box” is adopted for bearing packaging. However, the common packing method has a poor packing effect and poor versatility. In this study, a new biomimetic cobweb cushion is proposed to solve the problem of insufficient cushioning capacity of high-precision bearing cushion packaging pads. First, according to the nature of cobweb form, the cobweb cushion structure configuration is determined. Next, based on the structure of the cushion and the relationship between the parameters of radial thread and spiral thread, a mechanical and target optimization model is established. The stress nephogram of bearing and the cobweb cushion are analyzed under three drop heights of 381, 610, and 700 mm, in the finite element simulation software to ensure that the maximum bearings stress is not beyond the material yield strength. Via the 3D printing technology, a cobweb cushion shell cast is made. Drop tests of the bearing were performed, and the results were verified with the finite element simulation analysis. This research can provide technical support for the protection of high-precision bearings from accidental drops during transportation.

## 1. Introduction

Bearing is an essential component in modern mechanical equipment, its primary function is to support the mechanical rotating body, to reduce the mechanical load friction coefficient of the equipment in the transmission process, and its precision determines the working accuracy of mechanical products [1,2,3]. Among them, high-precision bearings are widely used in aerospace, military equipment, high-precision equipment and other fields due to their excellent performance, and the demand for high-precision bearings has grown exponentially [4,5]. The surface quality of bearings is a crucial factor that determines the performance of mechanical equipment. At present, studies have focused on the manufacturing process of high-precision bearings and various applications of machinery and equipment. However, the impact of various external factors on the surface quality of bearings has not been studied comprehensively. 

As a cushion carrier in the packaging structure, the cushioning material plays the main role of protecting high-precision bearings, which can decrease the influence of external shock and vibration, and absorb most of the energy of external shocks and vibrations suffered by high-precision bearings in the process of storage, transportation and turnover. Zhang Zhenyu proposed a cushion packing pad made of polydimethylsiloxane (PDMS) [6,7,8] for aviation bearings to improve the protection of bearings [9]. Yu Min et al. used 3D printing technology to fabricate thermoplastic polyurethane (TPU) cubic foam samples of different densities and sizes, and carried out mechanical compression tests, through comparative analysis, TPU materials have better cushioning performance [10,11], and medium-sized TPU cubic foam has the best mechanical properties [12]. Xu Ting et al. aimed at the design requirements of spacecraft for high-performance cushioning materials, the compression and cushioning properties of melamine foam [13,14] under different conditions were characterized by repeated compression cyclic loading, long-term constant pressure loading, long-term constant displacement loading and other loading methods [15]. Han Chong et al. compared the tensile strength, compressive strength and recycling feasibility of expanded polypropylene (EPP) and expanded polystyrene (EPS) [16,17] by testing and simulated transportation tests [18]. Bin Sun improved the outer packing of tapered roller bearings by adding expanded polyethylene (EPE) [19,20] pearl cotton between the outer ring and inner assembly and verified its protective effect [21]. Hu Tao et al. analyzed the impact of environmental factors on the cushioning performance of polyurethane (PU) [22,23] materials by observing the changes in appearance, static compression performance, compression set performance and infrared spectrum of polyurethane under the influence of temperature, humidity and other external environmental factors [24]. Shijie Wang et al. developed a bionic-inspired honeycomb column thin-walled structure (BHTS) by the biological structure of beetle’s elytra. Through the experiment, the energy absorption, maximum displacement and other parameters were analyzed, and it was found that the BHTS buffer interlayer can effectively protect the reinforced concrete slab structure from impact and explosion [25]. Taking advantage of the advantages of light weight and high strength of honeycomb structure, Zhenglei Yu et al. prepared the bionic lattice structure with memory alloy through the principle shape of structural bionics and found that the bionic lattice structure has excellent impact resistance and shape memory performance through quasi-static compression test and numerical simulation [26].

At present, the packing method of “plastic bag–buffer packing–packing paper box” is adopted for bearing packaging [27,28,29]. However, the common packing method has a poor packing effect and poor versatility. In this paper, we propose a method for a cobweb structure-based cushion made of HL-1029 material. The cushion concrete structure was designed by establishing and optimizing the mechanical model of the cobweb structure. The shell was obtained, and the bionic cushion was cast using 3D printing technology. Through the analysis of experimental data, the cushioning performance of cobweb structure cushion under three different drop heights were simulated in finite element software.

## 2. Biomimetic Research on Cobweb Configuration

### 2.1. Research on Cobweb Configuration

**Figure 1:**
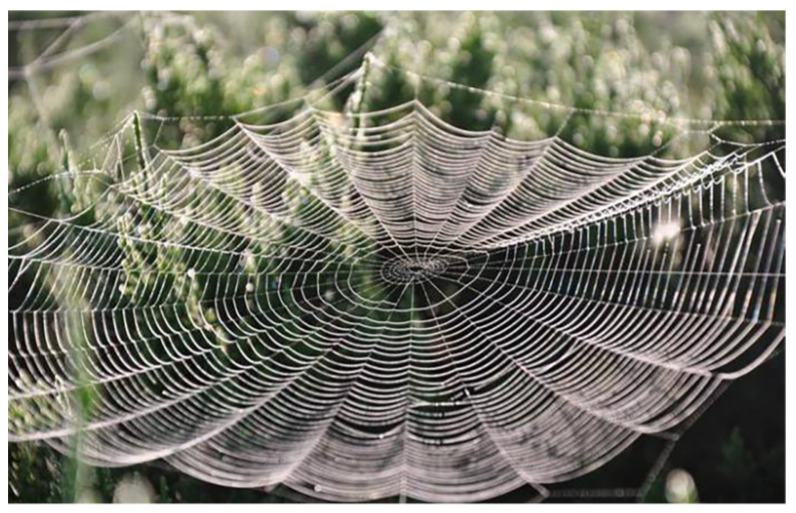
Flake web observed in nature.

The forms of cobwebs in nature are very diverse and can be broadly classified into 3 types: circular web with radial threads and spiral threads interwoven on a plane, flake web, and 3D irregular network. Among them, the flake web, containing a large predation area and robust wind resistance, is the most common, as shown in Figure 1.

**Figure 2:**
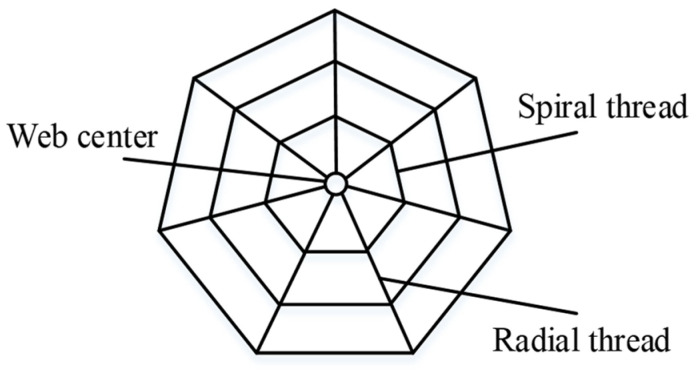
Simplified cobweb structure.

A flaky regular polygon was deduced as the shape of the web cushion by referring to the available literature and studying the shapes of different cobwebs observed in nature [30]. A simplified geometric structure containing only spiral threads, radial threads, and center was developed from the flaky regular polygon, as shown in Figure 2.

**Figure 3:**
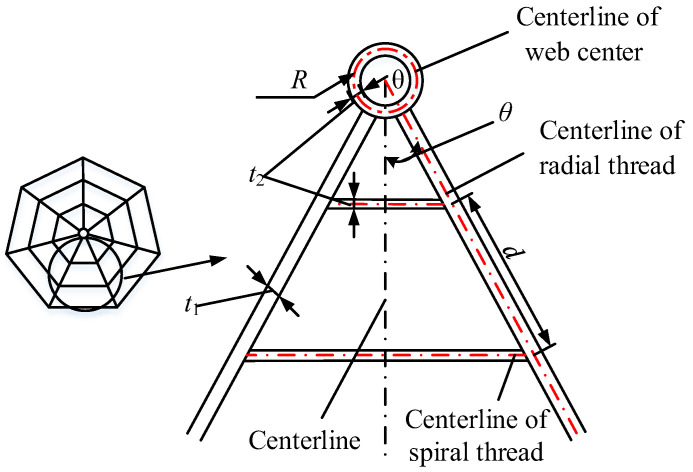
Structure parameters of cobweb.

As shown in Figure 3, a part of the simplified cobweb structure is taken as a micro-element for the study.

Where *R* is the radius of the centerline of the web center, *θ* is the included angle between the center line and the centerline of the radial thread, *d* is the distance of the centerline of the adjacent spiral thread, *t*_1_ is the width of the radial thread, and *t*_2_ is the width of the spiral thread.

### 2.2. Mechanical Model of Cobweb Structure Cushion

**Figure 4:**
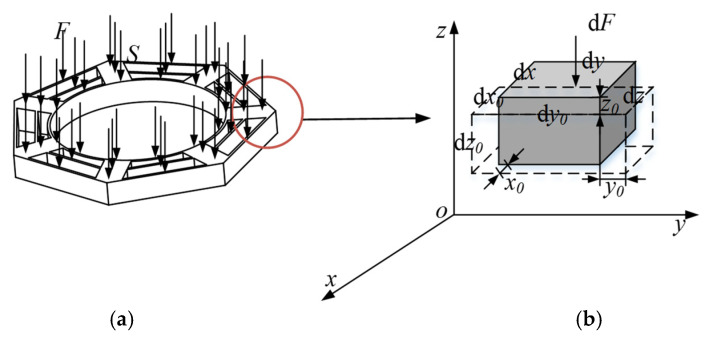
Overall cobweb structure diagram and Micro-element diagram of cobweb structure: (**a**) 3D structure diagram; (**b**) Micro-element diagram.

The cobweb structure diagram in Figure 2 is converted to a 3D structure with a certain thickness, in which the internal force surface surface is the upper surface of the cobweb structure, as shown in Figure 4a. Let the overall internal force applied to the cobweb structure be *F* and the upper surface area be *S*. For analysis, let us consider a micro-element with the dimension d*x*, d*y*, and d*z*, *x*_0_, *y*_0_, *and z*_0_ be the changes in length after the micro-element is forced in the three directions of *x*, *y*, and *z*, d*x*_0_, d*y*_0_, and d*z*_0_ be the final lengths after internal force deformation, respectively. Let d*F* be the internal force applied to the micro-element. The diagram of the micro-element is shown in Figure 4b.

According to the micro-element analysis, the overall normal stress of the cobweb structure is given by:

(1) σ=F/S

According to Equation (1), the larger the contact area, the smaller the stress. Therefore, the stress on the cobweb structure is smaller when the upper surface is larger. Further, shortening the total length of the cobweb structure cushion aids in reducing the waste of raw materials and overall costs. Therefore, maximizing the surface area and minimizing the total length are considered the optimization objectives.

Therefore, the optimization objectives can be formulated as:

(2) $$\min C=n_{1}n_{2}d(n_{2}\sin\theta +\sin\theta +1)+2R(n_{1}n_{2}\sin\theta +\pi )+\frac{1}{2\cos\theta }(n_{1}d-2n_{1}n_{2}t_{1})-\frac{n_{1}t_{2}}{2}$$

(3) $$\max S=n_{1}n_{2}t_{2}d\sin\theta (n_{2}+1)+\frac{n_{1}t_{1}t_{2}}{2\cos\theta }(1-\cos\theta -2n_{2})+n_{1}n_{2}(t_{1}d+2Rt_{2}\sin\theta )+2\pi Rt_{2}$$

where *C* is the total length of cobweb structure, *S* is the surface area of cobweb structure, *n*_1_ is the number of radial threads, and *n_2_* is the number of the spiral threads. 

We have:

(4) $$\theta =\pi /n_{1}$$

The cobweb cushion plays a protective role at the bottom of the bearing. Therefore, the inner connection radius of the outermost ring spiral thread of the cobweb structure cushion should be larger than the outer ring radius of the bearing.

(5) $$\left[R+n_{2}d+t_{2}/\left(2\cos\theta \right)\right]\cos\theta >r$$

where *r* is the outer ring radius of the bearing, the value of *r* is 90 mm.

The total width of all radial threads of the cobweb structure should be less than the length of the center of the web structure, and the width of the radial threads must be less than the side length of the outermost regular polygon of the outermost ring of the cobweb structure.

(6) $$2\left(R+t_{2}/2\right)\sin\theta >t_{1}$$

Considering the actual situation, the distance between the two adjacent spiral threads of the cobweb in nature is 5–15 mm. Therefore, the distance between the two spiral threads of the cobweb structure cushion is 5–15 mm [31].

(7) $$5<d\cos\theta -t_{2}<15$$

According to the literature [32], when the stiffness hg (stiffness refers to the ability of a material or structure to resist elastic deformation when subjected to force) ratio between the spiral thread and the radial thread is 1:10, the cushioning capacity is excellent. The height of the spiral thread and radial thread is the same in the cobweb cushion structure. Therefore, the width ratio between spiral and radial threads is 1: 10, written as follows:

(8) $$t_{1}=10t_{2}$$

The structure of a cobweb is a network structure, so the number of radial threads must be greater than or equal to three. Therefore, we get:

(9) $$n_{1}\geq 3$$

The centerline of the cobweb structure cushion passes through the inner ring of the bearing and the radial threads bear the cobweb structure cushion. To save material cost, the spiral thread width must be less than 1 mm.

(10) $$d_{2}/2<1$$

In summary, Equations (2) and (3) are set as optimization objectives and Equations (4)–(10) are set as constraints.

### 2.3. Optimization of Structural Parameters of Cobweb Bionic Cushion

**Figure 5:**
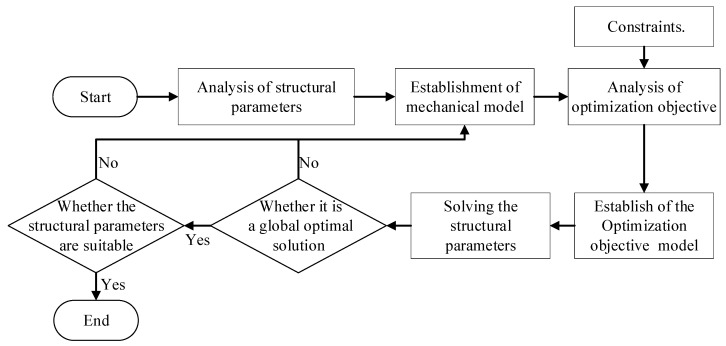
Flow chart of structure parameter solution.

We introduced the above optimization objectives and constraints into the mathematical modeling software and solved the structure parameters using the max-min method. The solution process is illustrated in Figure 5 below.

In this study, the outer ring diameter was 90 mm, and the inner ring diameter was 65 mm. To save the material cost, the radius of the centerline of the cobweb was equal to the radius of the inner ring of the test bearing, and the radius *R* of the centerline of the cobweb was 65 mm. The value of radial thread number *n*_1_, spiral thread number *n*_2_, spiral thread centerline spacing *d*, radial thread width *t*_1_, and spiral thread width *t*_2_ are 7, 2, 17, 20, and 2 mm, respectively.

### 2.4. Calculation of Cushion Thickness of Cobweb Structure

The mass of the test aero bearing was 2.2 kg, and its brittleness value Gc was 120 G [33]. Based on the product drop test height standard in ASTM D 4169 (Standard of American Society for Material Testing) [34], the drop height H was determined to be 381 mm.

HL-1029 is a low-viscosity, two-component, and highly transparent silicone potting adhesive that can be cured at room temperature. It is widely employed in the potting and sealing of precision electronic components and can be used as a raw material for bearing cushions. In the experiment, the test aviation bearing is placed flat on the HL-1029 silicone rubber cushion pad. The cushioned area *A* received by the aviation bearing is the contact area between the annular part between the outer ring and the inner ring and the cushion pad. By the software simulation, the value of *A* is found to be 0.0034 m^2^. The maximum stress acting on the cushion pad is given by:

(11) $$\sigma_{m}=\frac{{\mathrm{WG}}_{C}}{A}=0.777(\mathrm{Mpa})$$

where W is the bearing mass.

**Figure 6:**
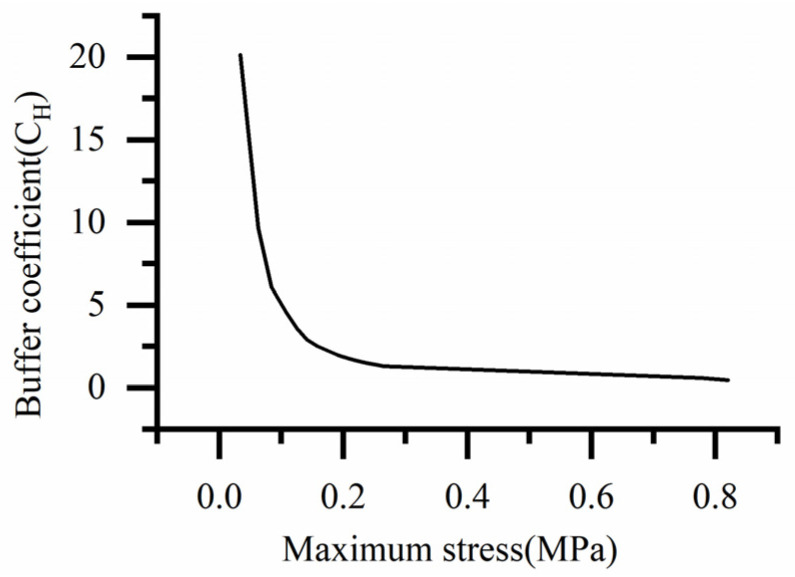
Stress-buffer coefficient curve of HL-1029 silica gel material.

The stress–buffer coefficient curve of the HL-1029 silica gel material is shown in Figure 6.

By fitting the curve in Figure 6, when the stress is 0.777 MPa, the corresponding buffer coefficient is 0.556 MPa. Using Equation (12) the thickness of the cobweb cushion is calculated to be 2 mm.

(12) $$T=\frac{C_{H}H}{G_{C}}=2(\mathrm{mm})$$

Therefore, the minimum thickness of the cobweb cushion was 2 mm to meet the buffering requirement. To more intuitively observe the damaged state of the cobweb cushion after the drop, prevent the bearing from causing greater damage due to the error of theoretical calculation and practical test, and improve the safety protection ability of the cobweb cushion structure for the bearing. We finally determined a safety factor is 9, and the thickness of the cobweb cushion structure is finally determined to be 18 mm.

## 3. Simulation

**Figure 7:**
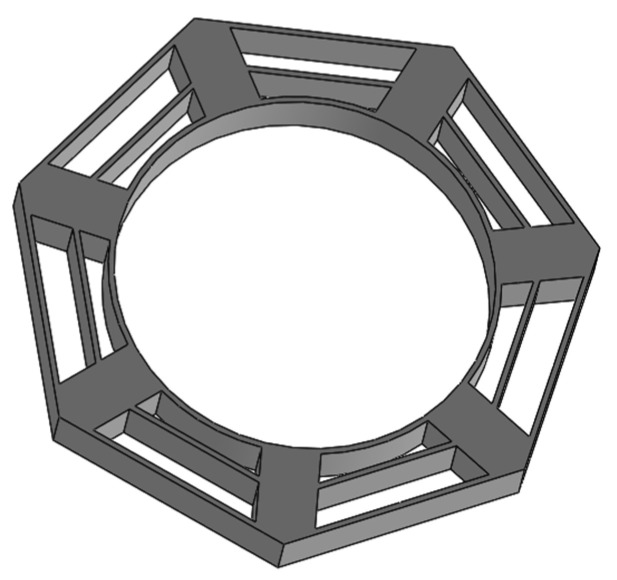
3D model of cobweb structure cushion.

Using the structural parameters obtained from the above analysis, we can draw a 3D model of the cobweb structure cushion in the 3D software (2018 X 64), as shown in Figure 7.

Based on the international drop test standards, the drop test heights was 381 mm. In addition, we also selected two drop test heights, 610 mm and 700 mm, as comparison groups. Therefore, the initial speeds were 2.773 m/s, 3.458 m/s, and 3.704 m/s, respectively, and the contact type of the bearing and the cobweb cushion were bonded. Bearing steel had a density of 7800 kg/m^3^, Young’s modulus of 210 GPa, and Poisson’s ratio of 0.3. The cobweb structure cushion was made of silicone HL-1029, with a density of 970 kg/m^3^ and a time length of 0.05 s. These were the parameters chosen for the tests. 

**Figure 8:**
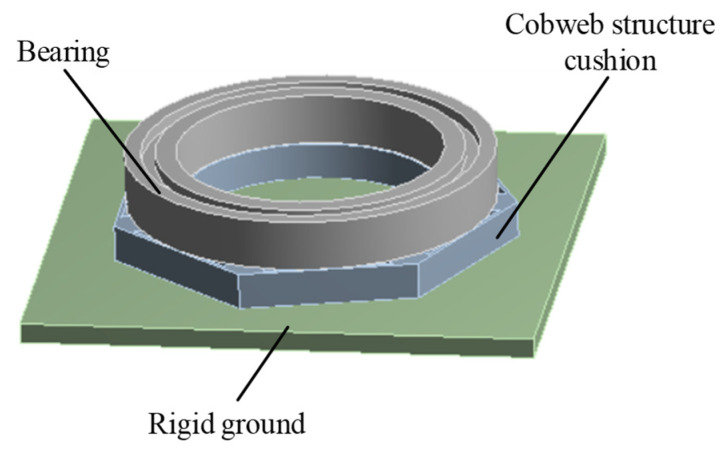
Drop state of bearing and cobweb structure cushion under finite element software environment.

Therefore, the display dynamics module was chosen as the solver of the simulation experiment. The entire test model included three parts: bearing, cobweb structure cushion, and rigid ground. To shorten the simulation solution time and simplify the simulation solution process, we adopt the simulation software to automatically divide the mesh, and the number of nodes divided is 9834 and the number of elements is 9496. In addition, the maximum factor of element quality is 0.99, which is very close to 1, indicating that the meshing meets the requirements. The drop state of the test bearing and cobweb structure cushion in the finite element environment is shown in Figure 8.

**Figure 9:**
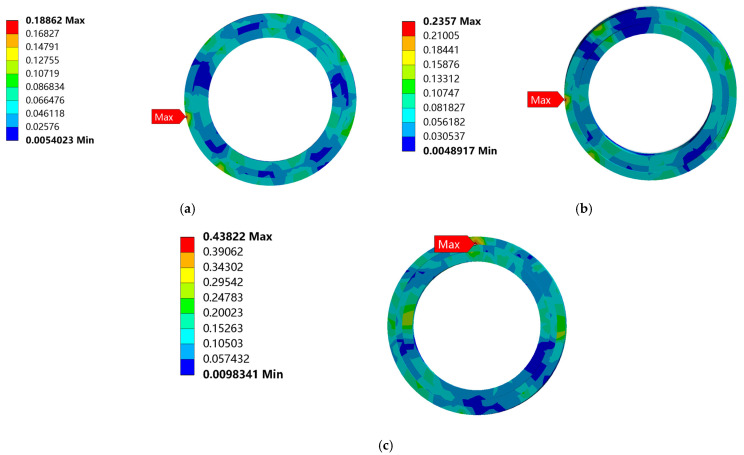
Equivalent stress nephogram of bearing drop: (**a**) Equivalent stress nephogram of bearing at the drop height of 381 mm; (**b**) Equivalent stress nephogram of bearing at the drop height of 610 mm; (**c**) Equivalent stress nephogram of bearing at the drop height of 700 mm.

**Figure 10:**
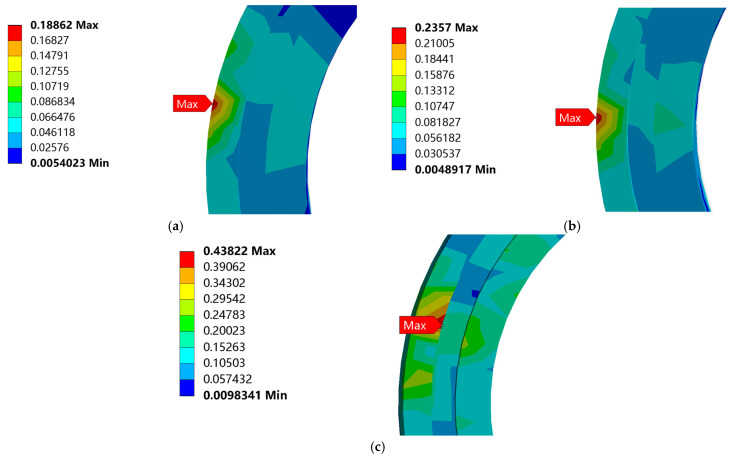
Enlarged nephogram of maximum equivalent stress at different drop heights: (**a**) Enlarged nephogram of maximum equivalent stress at the drop height of 381 mm; (**b**) Enlarged nephogram of maximum equivalent stress at the drop height of 610 mm; (**c**) Enlarged nephogram of maximum equivalent stress at the drop height of 700 mm.

The equivalent stress nephogram of the three drop heights is shown in Figure 9. From Figure 9, the largest equivalent stress appears at the bottom of the bearing outer ring. With an increase in the distance from the ground, the stress on the bearing gradually increases, and the bearing has no plastic deformation and damage even at maximum stress, as shown in Figure 10.

**Figure 11:**
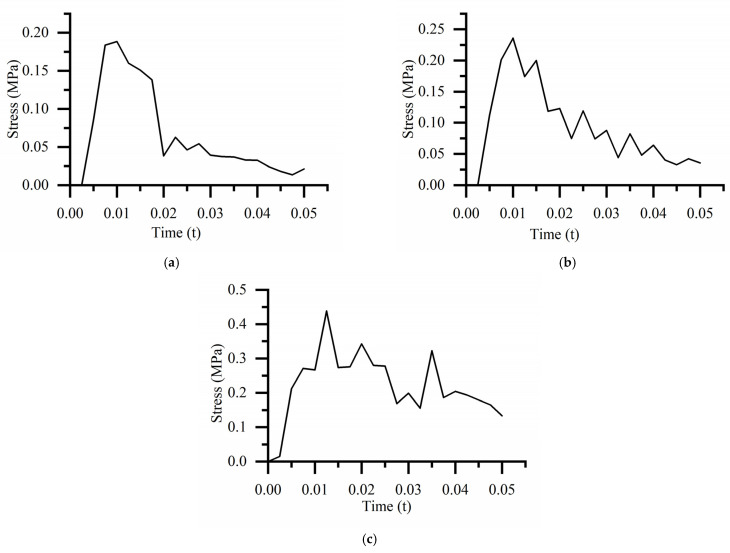
Equivalent stress curve at different drop heights: (**a**) Equivalent stress curve of bearing at the drop height of 381 mm; (**b**) Equivalent stress curve of bearing at the drop height of 610 mm; (**c**) Equivalent stress curve of bearing at the drop height of 700 mm.

The equivalent stress curve of the bearing is shown in Figure 11. For the drop height of 381 mm, the maximum equivalent stress is 0.189 MPa at 0.01 s. For the drop height of 610 mm, the maximum equivalent stress is 0.236 MPa at 0.01 s. For the drop height of 700 mm, the maximum equivalent stress is 0.438 MPa at 0.013 s.

With an increase in the drop height, the maximum stress on the bearing gradually increases. Because the limit range of the annealed yield strength of the test bearing materials is 353–382 MPa, the maximum stress at the three drop heights is far less than the yield strength. 

**Figure 12:**
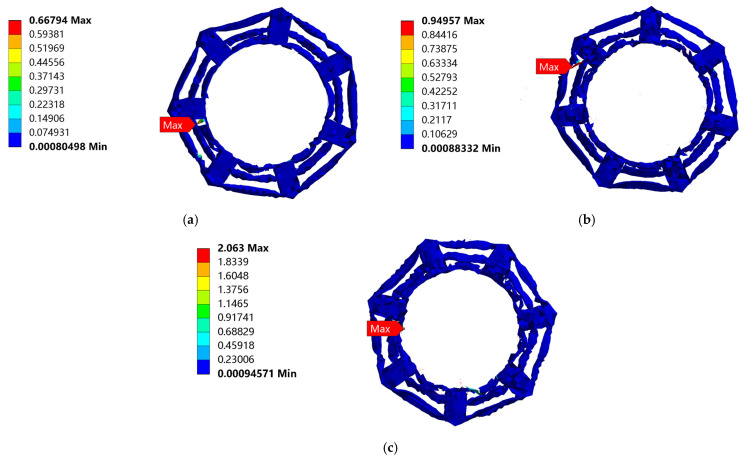
Equivalent stress nephogram of cobweb cushion structure drop: (**a**) Equivalent stress nephogram of cobweb cushion at the drop height of 381 mm; (**b**) Equivalent stress nephogram of cobweb cushion at the drop height of 610 mm; (**c**) Equivalent stress nephogram of cobweb cushion at the drop height of 700 mm.

**Figure 13:**
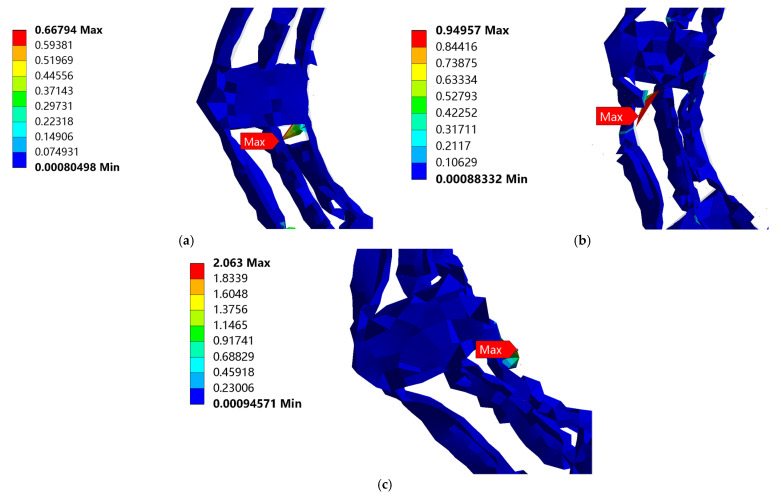
Enlarged nephogram of maximum equivalent stress when cobweb cushion is dropped from the three drop heights: (**a**) Enlarged nephogram of maximum equivalent stress when cobweb cushion is dropped from 381 mm; (**b**) Enlarged nephogram of maximum equivalent stress when cobweb cushion is dropped from 610 mm; (**c**) Enlarged nephogram of maximum equivalent stress when the cobweb cushion is dropped from 700 mm.

Figure 12 shows the cloud map of the equivalent stress on the cobweb cushion at the three drop heights. It can be seen from these images that the cobweb structure cushion is damaged, and the maximum equivalent stress occurs at the bottom of the cushion. The enlarged nephogram of maximum equivalent stress is shown in Figure 13.

**Figure 14:**
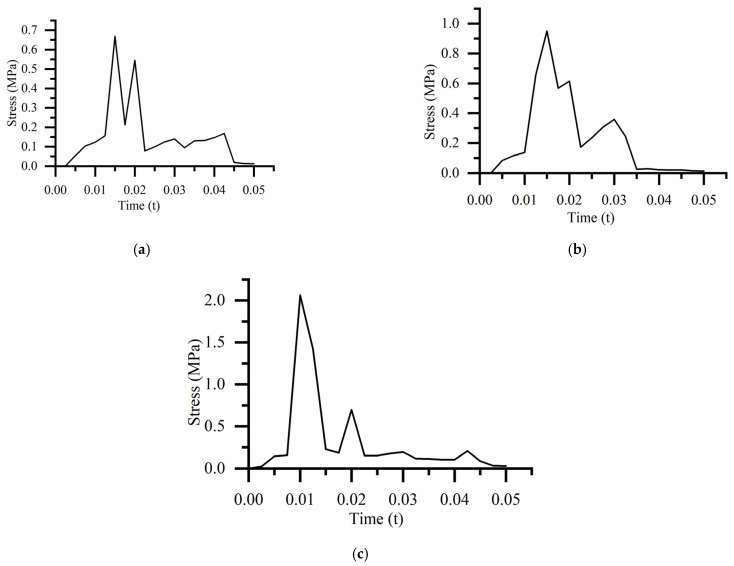
Equivalent stress curve of cobweb cushion at the different drop heights: (**a**) Equivalent stress curve of the cobweb cushion at the drop height of 381 mm; (**b**) Equivalent stress curve of the cobweb cushion at the drop height of 610 mm; (**c**) quivalent stress curve of cobweb cushion at the drop height of 700 mm.

The equivalent stress curve of the cobweb structure cushion is shown in Figure 14. For the drop height of 381 mm, the maximum equivalent stress was 0.668 MPa at 0.015 s. For the drop height of 610 mm, the maximum equivalent stress was 0.950 MPa at 0.015 s. For the drop height of 700 mm, the maximum equivalent stress was 2.063 MPa at 0.01 s. 

Accordingly, with an increase in the dropping height, the maximum stress of the cobweb structure cushion increases gradually, and the yield strength limit of HL-1029, which is used for the cobweb cushion, is 0.265 MPa, according to the constitutive relation, which is beyond the yield strength of the cobweb structure cushion. Therefore, the cushion was damaged.

**Figure 15:**
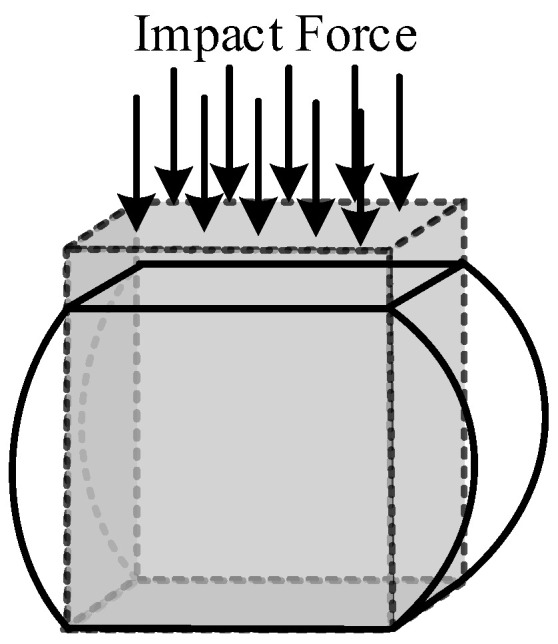
Radial threads “Bulging” phenomenon.

**Figure 16:**
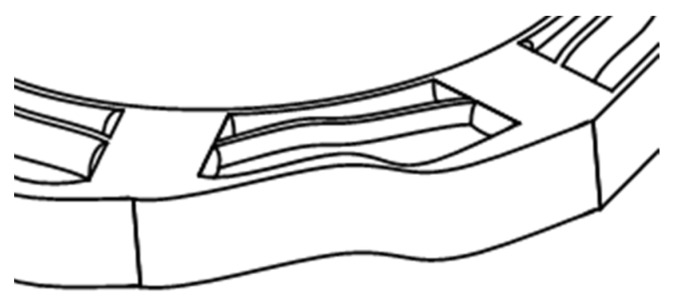
Spiral threads being squeezed phenomenon.

Through the analysis of the simulation results, we believe that when the cobweb cushion structure is impacted, the radial threads will be squeezed out to both sides due to the action of the impact force, resulting in the phenomenon of “Bulging”, as shown in Figure 15, due to the deformation of the radial threads, the spiral threads is subjected to pressure from the radial threads on both sides pointing to the center of the spiral threads which may cause the spiral threads to break due to too much extrusion force and absorb energy, as shown in Figure 16.

## 4. Preparation of the Cobweb Structure Cushion Material Cast

**Figure 17:**
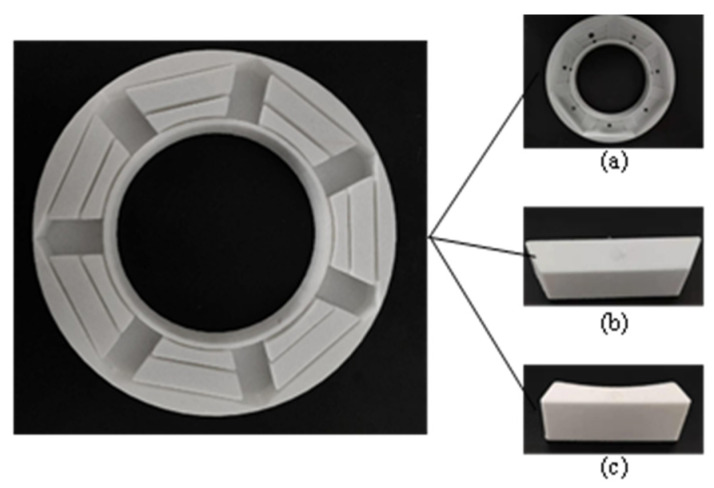
Cobweb structure cushion shell. (**a**) Shell base; (**b**) Part 1; (**c**) Part 2.

To obtain the cobweb cushion, we must make the shell of the cushion, develop the 3D model of the shell in 3D software, and print it using 3D printing technology [35,36]. The shell is now divided into three parts for convenience in printing and stripping. It is then assembled into a cast of HL-1029, as shown in Figure 17.

**Figure 18:**
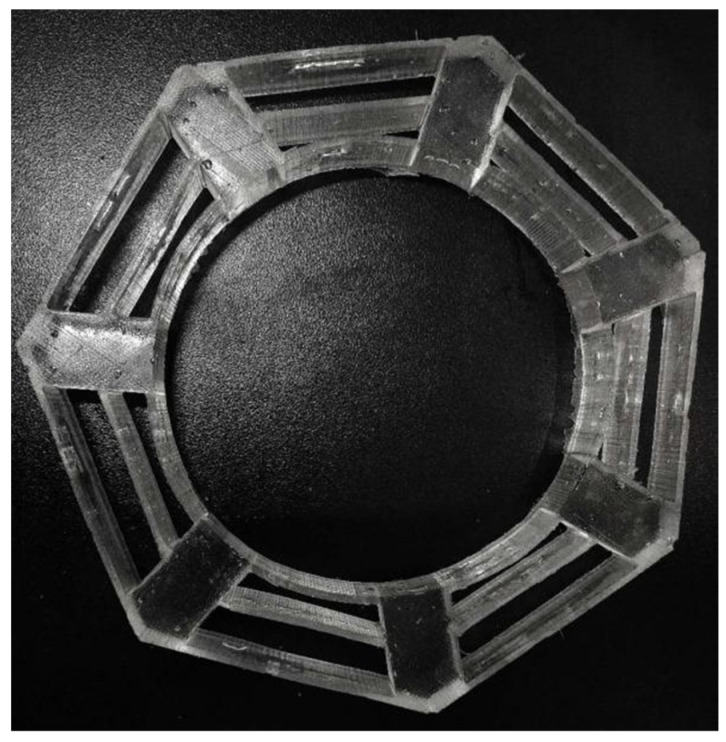
Cobweb structure cushion material object.

During casting, materials A and B were mixed evenly with a mass ratio of 1:1. The mixed material was slowly injected into the shell to prevent any bubbles. After allowing it to rest at room temperature for 24 h, it was unmolded to obtain the cobweb structure cushion, as shown in Figure 18. The casting conditions of the HL-1029 silicone are listed in Table 1.

## 5. Drop Test

### 5.1. Test Methods and Equipment Required

The standard drop height was obtained according to the relevant provisions in ASTM D 4169 (Standard of American Society for Material Testing). The bearing was placed above the cushion and glued to it. An acceleration sensor was placed on the surface of the test samples. Then, the bearing was placed on the table of the drop-testing machine and fixed with a fixed rod. The test table was raised to the set height to make the test piece drop freely. The impact acceleration of the bearing was recorded as it touched the ground. Next, the drop height was re-adjusted, and the test was repeated. Finally, the impact acceleration of the bearing at different drop heights was compared.

**Figure 19:**
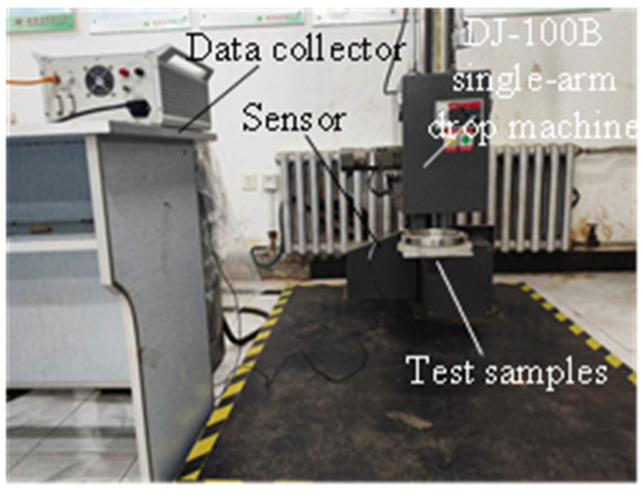
Drop test equipment.

**Figure 20:**
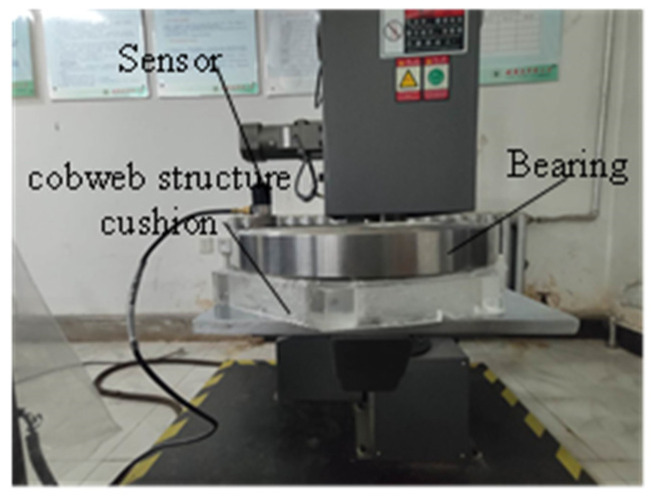
Test samples and sensors.

The equipment required for the drop test includes a data collector, acceleration sensor, and DJ-100B single-arm drop machine, as shown in Figure 19. The bearing and cobweb structure cushion is shown in Figure 20.

### 5.2. Analysis of the Test Results

**Figure 21:**
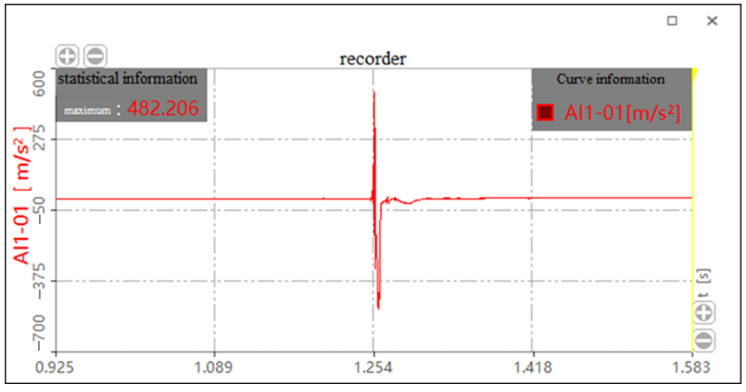
Impact acceleration curve with cushion for the drop height of 610 mm.

**Figure 22:**
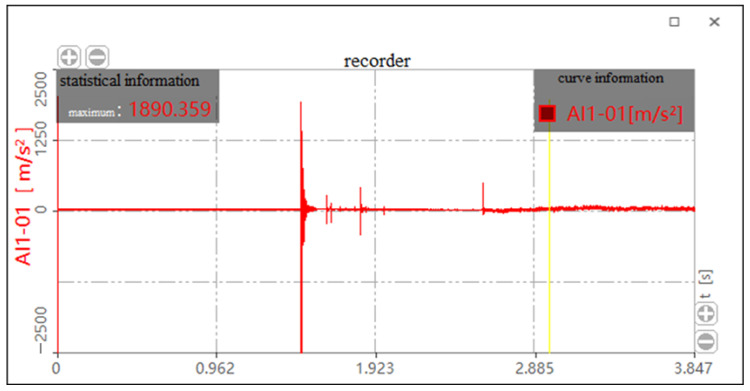
Impact acceleration curve without cushion for the drop height of 610 mm.

The test was divided into three groups according to the drop height of the test. Each group was tested several times. Finally, the most representative four data of each group from tests were considered for analysis. The bearing with and without the HL-1029 cushion package was tested for drop heights of 381 mm, 610 mm, and 700 mm, respectively. The thickness of the cobweb structure cushion was 18 mm. The impact acceleration curves with and without the cushion for the drop height of 610 mm are shown in Figure 21 and Figure 22, respectively.

**Figure 23:**
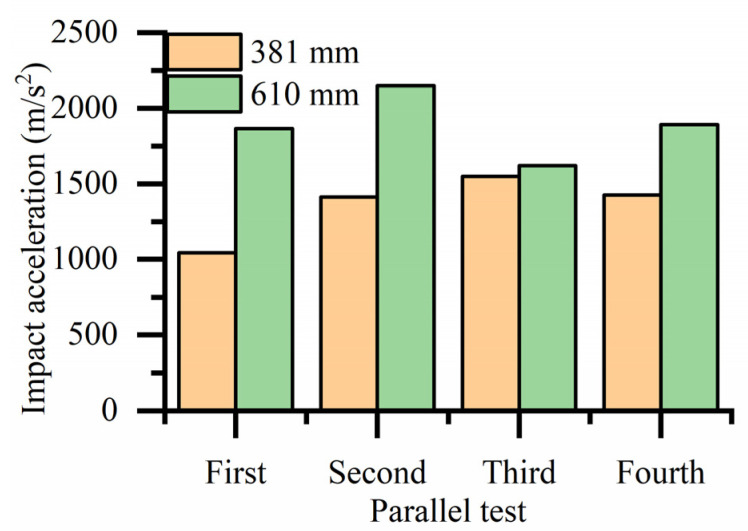
Histogram of impact acceleration of bearings drop at different heights without a cushion.

**Figure 24:**
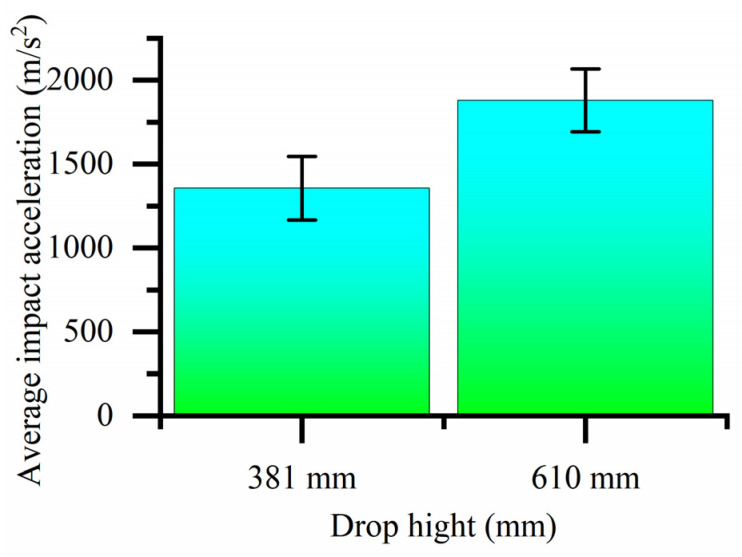
Histogram of average impact acceleration of bearings at different drop heights without cushion.

Because the impact acceleration generated by a 700 mm drop height is beyond the sensor range when there is no cushion, it cannot be tested. The bearing drop test data without a cushion, histogram of the bearing drop impact acceleration and histogram of average impact acceleration are shown in Table 2, Figure 23 and Figure 24, respectively. As shown in Figure 23, the maximum impact accelerations of the two drop heights without cushion are 1546.326 m/s^2^ and 2147.737 m/s^2^, respectively. It can be seen from the table that the average impact acceleration of the two drop heights without cushion is 1356.675 m/s^2^ and 1880.545 m/s^2^, respectively. All the above values exceed the brittle value of 120 G of the bearing. Therefore, the bearing is easily damaged without a cushion.

**Figure 25:**
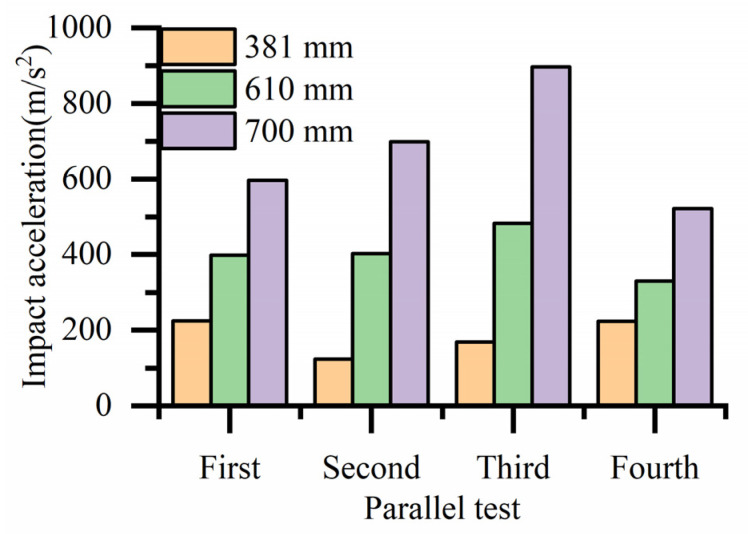
Histogram of impact acceleration of package drop at different heights with cushion.

**Figure 26:**
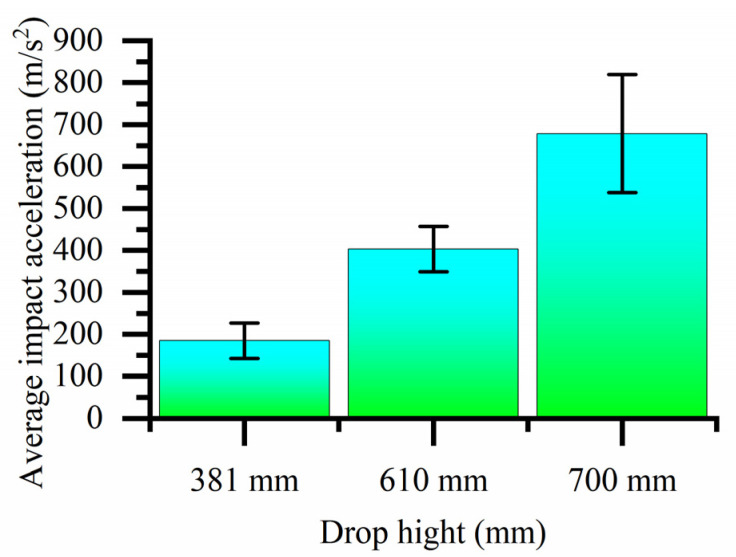
Histogram of average impact acceleration of bearings at different drop heights with cushion.

The bearing drop test data with the cobweb cushion, histogram of the bearing drop impact acceleration and histogram of average impact acceleration are shown in Table 3, Figure 25 and Figure 26, respectively. It can be seen that the impact acceleration of bearings with a cobweb structure cushion at three drop heights of 381, 610, and 700 mm is less than the brittleness value of 120 G.

**Figure 27:**
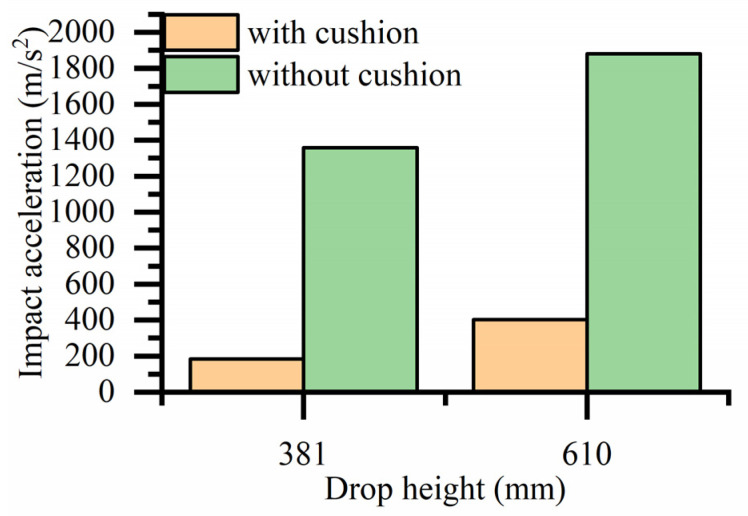
Histogram comparing the impact acceleration with or without cushion drop at the same drop height.

A histogram comparing the impact acceleration with and without the cobweb cushion at the same drop height is shown in Figure 27. The cobweb cushion can effectively reduce the impact acceleration of the bearing whenever it is dropped.

**Figure 28:**
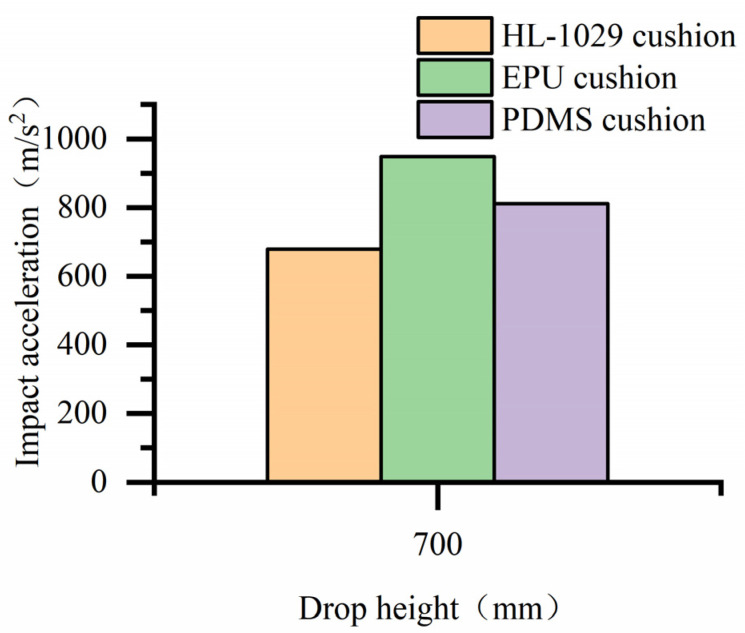
Histogram of impact acceleration with different materials cushion at the same drop height.

The impact acceleration of different materials cushions at a drop height of 700 mm is shown in Table 4 and Figure 28. It can be seen that the impact acceleration of the bearing with the cobweb structure cushion is 678.375 m/s^2^, the impact acceleration of the bearing with the expanded polyurethane (EPU) cushion is 948.555 m/s^2^, and the impact acceleration of the bearing with the Polydimethylsiloxane (PDMS) cushion is 810.953 m/s^2^. It can be seen from the histogram that the impact acceleration of the bearing is quite different when the three materials are cushioned, and the cobweb cushion with HL-1029 has the best cushioning performance.

## 6. Methods

In this paper, we analyze the cobweb structure cushion theoretically and experimentally, and the method’s process is as follows:

(1) A method of using organic silica gel as a cushion for aviation precision bearing packaging is proposed, and the structure of the cushion is designed based on a cobweb configuration.
(2) By increasing the structural parameters of the radial thread as a bearing force carrier, the minimum cushion thickness was calculated and designed. The theoretical calculation accuracy was verified by conducting simulation analysis.
(3) Using finite element software, drop simulations were carried out on a high-precision bearing packed with a biomimetic cobweb structure cushion, and stress cloud maps of the bearing and cushion were obtained.
(4) The biomimetic cobweb structure cushion casting was carried out using a 3D printed cobweb cushion shell. The impact acceleration of the bearing was obtained using a drop test.

## 7. Conclusions

A new biomimetic cobweb cushion is proposed to solve the problem of insufficient cushioning capacity of high-precision bearing cushion packaging pads. the results can be summarized as follows:

(1) The structural parameters of the cobweb structure cushion were solved by the optimization equation, and then the thickness is 18 mm according to the actual situation.
(2) Analysis and processing of the drop test results show that at a drop height of 381 mm and 610 mm, the impact acceleration of the bearing with a cobweb structure cushion is reduced by 86% and 78%, respectively, compared with the without cobweb structure cushion. At a drop height of 700 mm, the impact acceleration of the bearing with the HL-1029 cushion is reduced by 28% and 16% compared with the EPU cushion and PDMS cushion, respectively.
(3) The test results showed that the biomimetic cobweb structure cushion could provide robust protection for high-precision bearings.

## Figures and Tables

**Table 1:** Casting conditions of HL-1029 silicone.

Parameter	Value
Mixing ratio A:B	1:1
Gel time	8–12 h (25 °C)
The available time of exposure to air	1 h (25 °C, mixture 100 g)
Hardening time	24 h (25 °C)

**Table 2:** Drop test data of bearing without cushion.

Drop Height (mm)	a1 (m/s^2^)	a2 (m/s^2^)	a3 (m/s^2^)	a4 (m/s^2^)	Average Impact Acceleration (m/s^2^)
381	1041.088	1412.530	1546.326	1426.754	1356.675
610	1864.119	2147.737	1619.964	1890.359	1880.545
700	/	/	/	/	/

**Table 3:** Drop test data of HL-1029 cushion bearing packaging.

Drop Height (mm)	a1 (m/s^2^)	a2 (m/s^2^)	a3 (m/s^2^)	a4 (m/s^2^)	Average Impact Acceleration (m/s^2^)
381	224.055	122.693	168.220	223.283	184.563
610	398.359	401.640	482.206	329.835	403.009
700	596.466	698.199	896.778	522.056	678.375

**Table 4:** Drop test data of 700 mm drop height with different materials cushion.

Different Materials Cushions	Impact Acceleration (m/s^2^)
HL-1029	678.375
EPU	948.555
PDMS	810.953

## Data Availability

Data will be made available on request.
